# Development and Validation of a Novel Stability-Indicating RP-HPLC Method for the Simultaneous Determination of Halometasone, Fusidic Acid, Methylparaben, and Propylparaben in Topical Pharmaceutical Formulation

**DOI:** 10.3797/scipharm.1301-21

**Published:** 2013-02-25

**Authors:** Nishant Goswami, V. Rama Mohan Gupta, Hitesh A. Jogia

**Affiliations:** 1 Analytical Research and Development, Integrated Product Development, Dr. Reddy’s Laboratories Ltd., Bachupally, Hyderabad-500 072, India.; 2 Singhania University, Pacheri Bari, Dist. Jhunjhunu, Rajasthan-333 515, India.; 3 Pulla Reddy Institute of Pharmacy, Dundigal, Jinnaram, Hyderabad, A.P.-502 313, India.

**Keywords:** Halometasone Monohydrate, Methylparaben, Propylparaben, Method validation, Forced degradation, Topical formulation, Assay, HPLC

## Abstract

A stability-indicating reversed-phase high-performance liquid chromatography (RP-HPLC) method was developed for the simultaneous determination of halometasone, fusidic acid, methylparaben, and propylparaben in topical pharmaceutical formulation. The desired chromatographic separation was achieved on an Agilent Zorbax CN (Cyano), 5 μm (250 × 4.6 mm) column using gradient elution at 240 nm detector wavelength. The optimized mobile phase consisted of a mixture of 0.01 M phosphate buffer and 0.1% orthophosphoric acid, pH-adjusted to 2.5 with an ammonia solution as solvent-A and acetonitrile as solvent-B. The developed method separated halometasone, fusidic acid, methylparaben, and propylparaben in the presence of known impurities/degradation products. The stability-indicating capability was established by forced degradation experiments and separation of known and unknown degradation products. The developed RP-HPLC method was validated according to the International Conference on Harmonization (ICH) guidelines. This validated method was applied for the simultaneous estimation of HM, FA, MP, and PP in commercially available cream samples. Further, the method can be extended for the estimation of HM, FA, MP, and PP in various commercially available dosage forms.

## Introduction

Halometasone (HM) is a corticosteroid. Corticosteroids act by the induction of lipocortins which prevent the formation of prostaglandins and leukotrienes. Both prostaglandins and leukotrienes are mediators which lead to inflammation. Halometasone acts by blocking their production, thus acting as an anti-inflammatory agent. It is available as a cream for topical use and used to treat chronic psoriasis vulgaris [1] and non-infected acute eczematous dermatoses [2]. Its empirical formula is C_22_H_27_CIF_2_O_5_ and its structure is shown in Fig. 1.

Fusidic acid (FA) is a bacteriostatic antibiotic used in the treatment of primary and secondary skin infections caused by sensitive strains of S. aureus, Strepto cocci species, and C. Minutissimum. Fusidic acid is the steroidal antibiotic used to treat Gram positive infections. It acts by preventing the translocation of peptidyl tRNA. Resistant mutants are easily selected, even during therapy, and therefore fusidic acid is usually administered in combination with another antibiotic. This helps reduce the risk of selecting resistant mutants. To survive, the fusidic acid-resistant mutants become resistant to the antibiotic given in combination [4, 5]. The FA raw materials, cream and eye drops, have also been described in the British Pharmacopoeia in 2005 (BP 2005) [6]. Its empirical formula is C_31_H_48_O_6_ and its structure is shown in Fig. 1.

Liquid preparations are particularly susceptible to microbial growth because of the nature of their ingredients. Such preparations are protected by the addition of preservatives that prevent the alteration and degradation of the product formulation [6].

Methylparaben (MP) and propylparaben (PP) have been used for the preservation of both active substances in the dose formulation and the structures of both are depicted in Tab. 1. The finished product release specifications should include an identification test and a content determination test with acceptance criteria and limits for each antimicrobial preservative present in the formulation [7]. The finished product shelf-life specification should also include an identification test and limits for the antimicrobial preservatives present [9]. Hence their (MP and PP) antimicrobial and antifungal properties make them an integral part of the product formulation. This encourages the development of a new stability-indicating method for the simultaneous estimation of all compounds (HM, FA, MP, and PP) to provide driving force in today’s pharmaceutical industry.

The literature survey revealed that there are some analytical methods reported for the qualitative and quantitative determination of FA either individually, like the visible spectrophotometric method, HPTLC, or in combination with other drugs by HPLC, which also reported on biological fluids [9–12].

The combination of HM and FA is not official in any pharmacopoeia. So far, no RP-HPLC stability-indicating method has been reported for the rapid simultaneous determination of HM, FA, MP, and PP in topical pharmaceutical formulation.

Therefore, it is necessary to develop a new rapid and stability-indicating method for the simultaneous determination of four compounds (HM, FA, MP, and PP) in topical pharmaceutical formulation. The proposed method is able to separate HM, FA, MP, and PP from each other and from other known impurities/degradation products. As a result, this method was validated according to the ICH guidelines [13] and successfully applied for the separation and quantification of all compounds of interest in topical pharmaceutical formulation.

## Experimental

### Chemicals, reagents and samples

The drug product, placebo solution, working standards, and reference standards were provided by Dr. Reddy’s laboratories Ltd., Hyderabad, India. HPLC grade acetonitrile and methanol were obtained from J. T. Baker (NJ, USA). GR grade potassium dihydrogen phosphate, GR grade orhtophosphoric acid, and GR grade ammonia solution were obtained from Merck Ltd. (Mumbai, India). The 0.22 μm nylon membrane filter and nylon syringe filters were purchased from Pall Life Science Limited (India). A 0.22 μm PVDF syringe filter was purchased from Millipore (India). High-purity water was generated by using the Milli-Q Plus water purification system (Millipore^®^, Milford, MA, USA).

### Equipments

The Waters HPLC system (Waters 2695 Alliance Separation Module) (eg. Waters Milford, USA), consisted of a binary solvent manager, sample manager, and PDA (photodiode array) detector. System control, data collection, and data processing were accomplished using Waters Empower TM- 2 chromatography data software. Photostability studies were carried out in a photostability chamber (SUN TEST XLS+, Atlas, USA). Thermal stability studies were performed in a dry air oven (Merck Pharmatech, Hyderabad, India).

### Chromatographic conditions

The chromatographic condition was optimized using the Agilent Zorbax CN, 5 μm (250 × 4.6 mm) column. The mobile phase involved a variable composition for solvent-A (solution containing 0.01M phosphate buffer (KH_2_PO_4_) and 1mL orthophosphoric acid per liter of water, adjusted to pH 2.5 with the ammonia solution) and acetonitrile was used as solvent-B. Solvents-A and B were filtered through a 0.22 μm nylon membrane filter and degassed under vacuum prior to use. The separation of HM, FA, MP, PP, and all impurities was achieved by gradient elution using solvent-A and B. A mixture of the buffer and acetonitrile in the ratio of 50:50 (v/v), respectively, was used as a diluent. The gradient program was as follows: time (min)/%B; T_0.01_/35, T_10_/35, T_15_/60, T_20_/60, T_22_/35, T_25_/35, at a flow rate of 1.0 mL/min at 25°C (column oven) temperature, detection wavelength 240 nm. Under these conditions, the backpressure in the system was about 2,000 psi. The stress-degraded samples and the solution stability samples were analyzed using a PDA detector covering the range of 200–400nm.

### Standard solution preparation

Standard solution was prepared by dissolving standard substances in diluent to obtain a solution containing 12.5 μg/mL of halometasone, 500 μg/mL of fusidic acid, 37.5 μg/mL of methylparaben, and 3.75 μg/mL of propylparaben.

### Sample solution preparation

An accurately weighed 5 gm of sample solution was taken into the 100 mL volumetric flask. About 70 mL of tetrahydrofuran was added to this volumetric flask and sonicated in an ultrasonic bath for 20 min. This solution was then diluted up to the mark with tetrahydrofuran and mixed well. Then 5 mL of this solution was further diluted to 10 mL with diluent and it was then filtered through a 0.22 μm PVDF syringe filter and the filtrate was collected after discarding the first few milliliters.

### Placebo (other substances without HM, FA, MP, and PP) solution preparation

An accurately weighed 5 gm of placebo solution was taken into the 100 mL volumetric flask. About 70 mL of tetrahydrofuran was added to this volumetric flask and sonicated in an ultrasonic bath for 20 min. This solution was then diluted up to the mark with tetrahydrofuran and mixed well. Then 5 mL of this solution was further diluted to 10 mL with diluent and it was then filtered through 0.22 μm PVDF syringe filter and the filtrate was collected after discarding the first few milliliters.

## Results and Discussion

### Optimization of chromatographic conditions

The primary target in developing this HPLC method was to achieve the simultaneous determination of HM, FA, MP, and PP in topical formulation under common chromatographic conditions; this method is applicable for routine quality control of products in pharmaceutical and cosmetic industries.

The optimization of the stationary phase and mobile phase was done simultaneously. The stationary phases such as the Hypersil BDS C18 and Luna C8 were tried with mobile phases such as glacial acetic acid, ammonium phosphate buffer (pH 4.5), and triethylamine buffer (pH 2.5), and their composition with methanol, acetonitrile, and tetrahydrofuran were tried but problems such as co-elution of FA and placebo peaks, peak broadening of MP, and placebo peak interferences etc. were observed. Good chromatography was observed using the Agilent Zorbax CN, 5 μm (250 × 4.6 mm) column. A mixture of 0.01M phosphate buffer (KH2PO4) in 0.1% orthophosphoric acid, pH-adjusted to 2.5 with the ammonia solution, and acetonitrile was used as solvent-B. The wavelength was selected by injecting a known concentration of each of HM, FA, MP, and PP into the HPLC with a PDA detector and evaluating the UV spectra of each component. A common wavelength for the simultaneous determination of all components was selected as 240 nm by overlaying the spectra and wavelengths at which all components had significant absorbance. Other chromatographic parameters were finalized such as the flow rate of 1.0 ml/min, column temperature of 25°C, and injection volume (10 μL).

The extraction of active components from the semisolid sample matrix with acceptable recovery was a very critical aspect for sample preparation and was achieved by choosing the right diluent (solvent) in the following manner. Tetrahydrofuran was selected as the solvent for dispersion and dissolving the active components in the sample matrix and the diluent was selected as the solvent for the final dilution of the sample preparation.

**Fig. 2:**
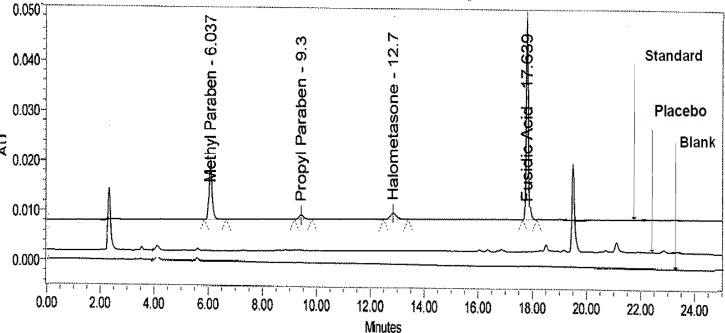
Typical overlay chromatogram of the blank and Placebo and standard preparation

Based on the above experimental data, the chromatographic separation was finalized by following the gradient program time (min)/%B; T_0.01_/35, T_10_/35, T_15_/60, T_20_/60, T_22_/35, T_25_/35, at a flow rate of 1.0 mL/min at 25°C (column oven) temperature, detection wavelength 240 nm with 10 μL injection volume. By using the above chromatographic conditions and diluents; the standard, sample, and placebo preparation were prepared and injected into the HPLC with the developed parameters (Fig.-2).

## Analytical Method validation

After satisfactory development of the method, it was subjected to method validation as per ICH guidelines [14, 15]. The method was validated to demonstrate that it is suitable for its intended purpose by the standard procedure to evaluate the adequate validation characteristics (system suitability, accuracy, precision, linearity, robustness, solution stability, filter compatibility, and stability-indicating capability).

### Precision

#### Instrument precision: (Suitability of system)

System suitability parameters were measured so as to verify the system performance. System precision was determined on six replicate injections of the standard preparation. All important characteristics including % RSD, tailing factor, and theoretical plate number were measured.

#### Method precision: (Repeatability)

The precision of the assay method was evaluated by carrying out six independent determinations of HM, FA, MP, and PP (12.5 μg/mL of HM, 500 μg/mL of FA, 37.5 μg/mL of MP, and 3.75 μg/mL of PP) test samples against the qualified working standard.

#### Intermediate precision: (Reproducibility)

The purpose of this study was to demonstrate the reliability of the test results with variations. The reproducibility was checked by analyzing the samples by a different analyst using a different chromatographic system and column on a different day.

### Specificity

Specificity is the ability of the method to measure the analyte response in the presence of its potential degradants and placebo matrix. In the present study, injections of the blank, placebo, and standard were performed to demonstrate the interference with the elution of HM, FA, MP, and PP. These results demonstrate that there was no interference from the other compounds which, therefore, confirms the specificity of the method (Figure 2).

**Fig. 3:**
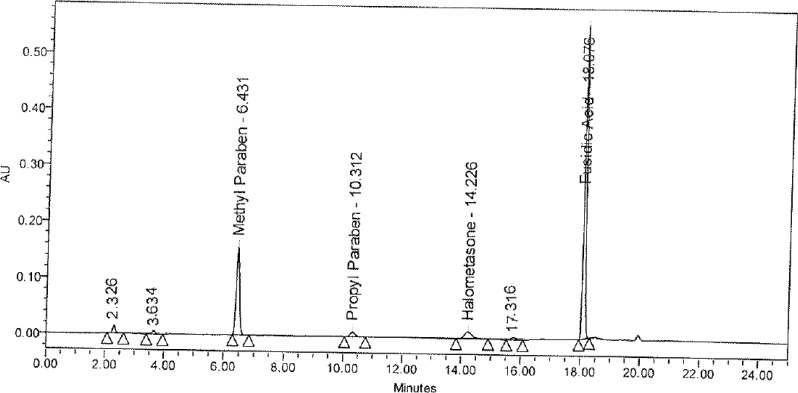
Typical chromatogram of the base-stressed sample

**Fig. 4:**
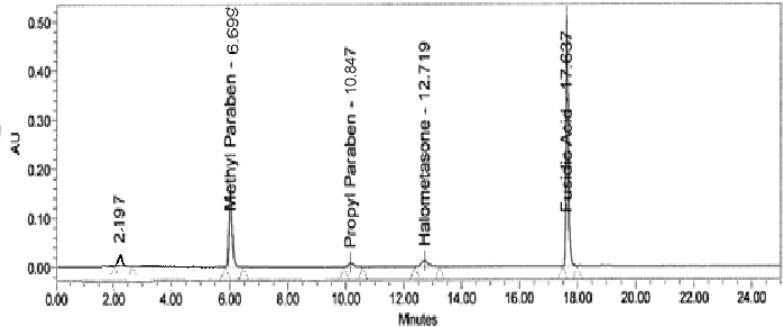
Typical chromatogram of the peroxide-stressed sample

Forced degradation studies of the drug product were also performed to evaluate the stability-indicating property and specificity of the proposed method. The solutions of the drug product and placebo were exposed to acid hydrolysis (0.1N HCl at 60 °C for 20 min), base hydrolysis (0.1 N NaOH at 60 °C for 20 min), oxidation (3% H_2_O_2_ at room temperature for 1 h), hydrolytic (water at 60 °C for 15 min), thermal (105 °C for 1h), and photolytic degradation (drug product exposed to visible light for 240 h resulting an overall illustration 1.2 million lux hours and UV light for 250 h resulting in an overall illustration 200 w h/m2 at 25 °C). Significant degradation was observed during the hydrolytic, base hydrolysis, thermal, photolytic, and oxidative degradation (Figures 3 and 4). The peak purity test was carried out for the HM, FA, MP, and PP peaks by using the PDA detector in the stress samples. The purity of all four substances was unaffected by the presence of the degradation products, and, thus confirms the stability-indicating power of the developed method. The summary data of the stress study is shown in Table 3.

### Linearity

**Fig. 5:**
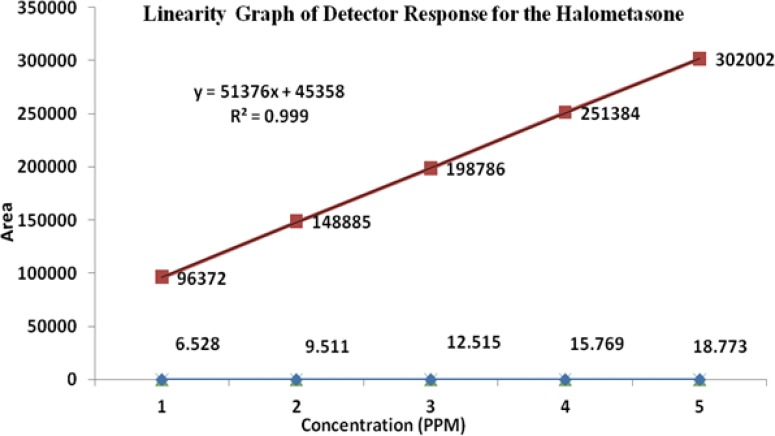
Linearity graph of Halometasone

**Fig. 6:**
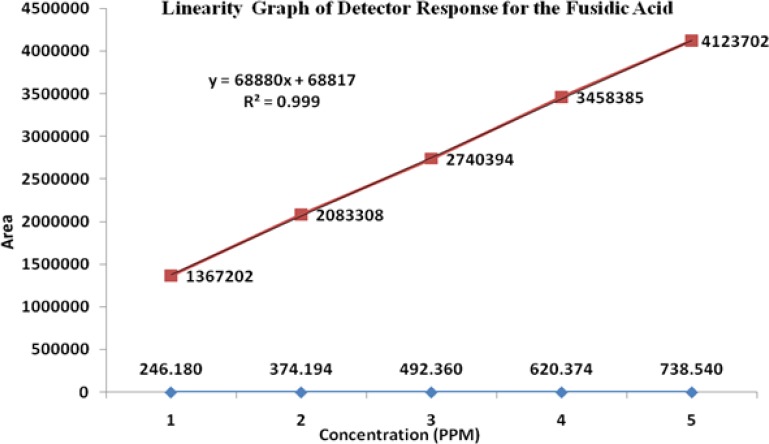
Linearity graph of Fusidic acid

**Fig. 7:**
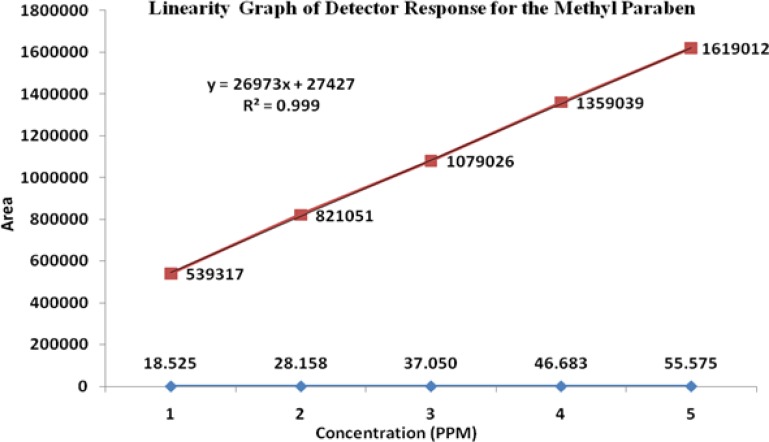
Linearity graph of Methylparaben

**Fig. 8:**
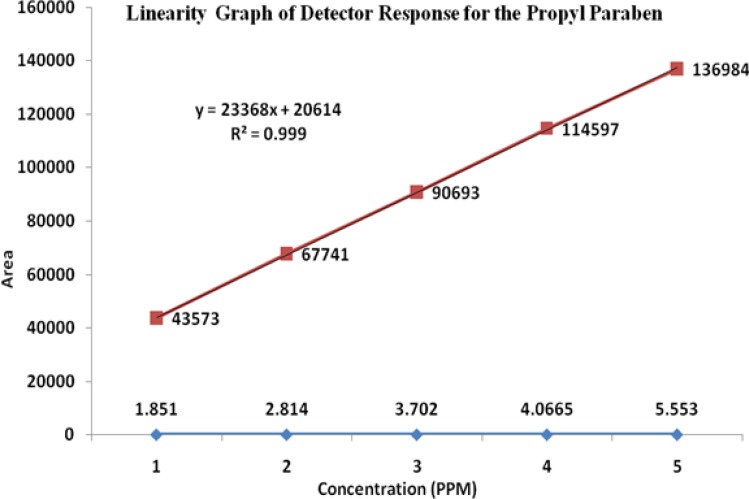
Linearity graph of Propylparaben

The linearity of an analytical method is its ability to elicit test results that are directly, or by a well-defined mathematical transformation, proportional to the concentration of the analyte in a sample covering the range of 50, 75, 100, 125, and 150% of the normal limit concentration. The nominal concentrations of the standard and test solutions for HM, FA, MP, and PP were 12.5μg/mL, 500μg/mL, 37.5μg/mL, and 3.75μg/mL, respectively. The correlation coefficients, slopes, and Y-intercepts of the calibration curve were reported (Table 4 and Fig. 5–8) and the results show that an excellent correlation existed between the peak area and the concentration of HM, FA, MP, and PP.

### Accuracy

The accuracy of an analytical method is the closeness of test results obtained by that method compared with the true values. To confirm the accuracy of the proposed method, recovery experiments were carried out by the standard addition technique. The accuracy of the method was carried out by adding known amounts of each drug corresponding to three concentration levels; 50, 75, 100, 125, and 150% of the actual concentration along with the excipients in triplicate. The percentage recoveries of HM, FA, MP, and PP at each level and each replicate were determined. The percentage recoveries for all four components were calculated (Table-5). The percentage mean recovery of HM, FA, MP, and PP from the formulation varied from 97.6 to 102.0 %, indicating that the developed method was accurate for the determination of HM, FA, MP, and PP in the pharmaceutical formulation.

### Robustness

The robustness of the method was evaluated during development by making small, but deliberate changes to the method parameters. The variables evaluated in the study were pH of the mobile phase buffer (± 0.2), column temperature (± 5°C), and flow rate (± 0.2 ml/min), and system suitability parameters such as % RSD, retention time, tailing factor, and theoretical plates of HM, FA, MP, and PP standard were studied. In all of the deliberately varied chromatographic conditions, the system suitability parameters met the acceptance criteria (Table 6). Thus, the method was found to be robust with respect to variability in applied conditions.

### Stability of the analytical solution

The solution stability of FM, FA, MP, and PP in the assay method was investigated by leaving the standard and sample solutions in tightly capped volumetric flasks at room temperature for 24 hours. The same sample solutions were analyzed at the end of the study period against freshly prepared standard solutions. The variability in the assay of all four substances was within ± 3% during solution stability. The results from the solution stability experiments confirmed that the sample solution and standard solutions were stable up to 24 hr.

### Filter compatibility

The spiked sample solution filtered through different types of membrane syringe filters (Centrifuged, Glass, Nylon, PVDF and Teflon) were injected into the HPLC. The % difference was calculated against the centrifuged sample solution. The results show that the % difference against the centrifuged is within the limit ± 0.05.

## Conclusion

A gradient RP-HPLC method was successfully developed for the simultaneous estimation of halometasone, fusidic acid, methylparaben, and propylparaben in topical pharmaceutical formulation. The developed method is selective, precise, accurate, linear, filter compatible, and robust. The forced degradation data proved that the method is specific for the analytes and free from the interference of the placebo / known impurities / and degradation products. Moreover, it may be applied for the individual and simultaneous determination of halometasone, fusidic acid, methylparaben, and propylparaben compounds in a pharmaceutical drug product and substance. Also, it can be utilized for the determination of an assay, blend uniformity, and content uniformity of pharmaceutical products.

## Figures and Tables

**Tab. 1.** Chemical structure of Halometasone, Fusidic acid, Methylparaben, and Propylparaben

**Name**	**Chemical Name**	**Chemical Structure**
Halometasone	(6α,11β,16α)-2-chloro-6,9-difluoro-11,17,21-trihydroxy-16-methyl-pregna-1,4-diene-3,20-dione	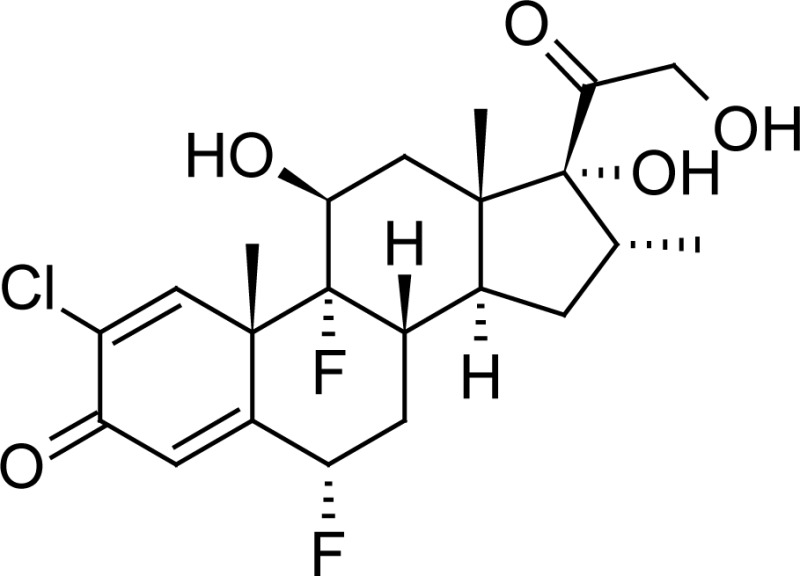
Fusidic acid	(2*Z*)-2-[(3α,4α,5α,8α,9β,11α,13α,-14β,16β,17*Z*)-16-(acetyloxy)-3,11-dihydroxy-4,8,10,14-tetramethylgonan-17-ylidene]-6-methylhept-5-enoic acid	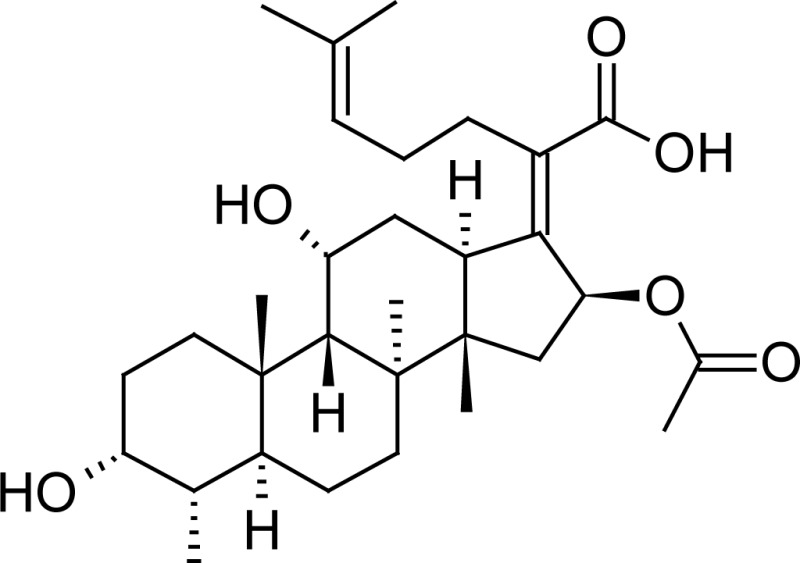
Methylparaben (MP)	methyl 4-hydroxybenzoate	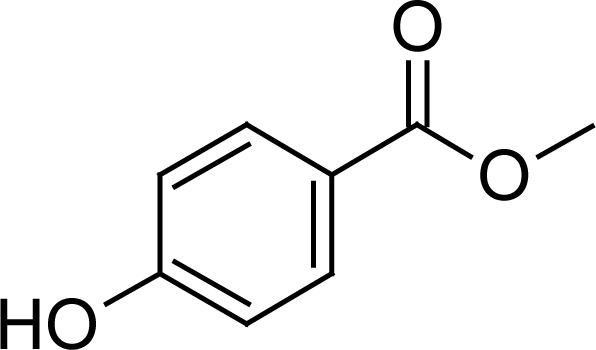
Propylparaben (PP)	propyl 4-hydroxybenzoate	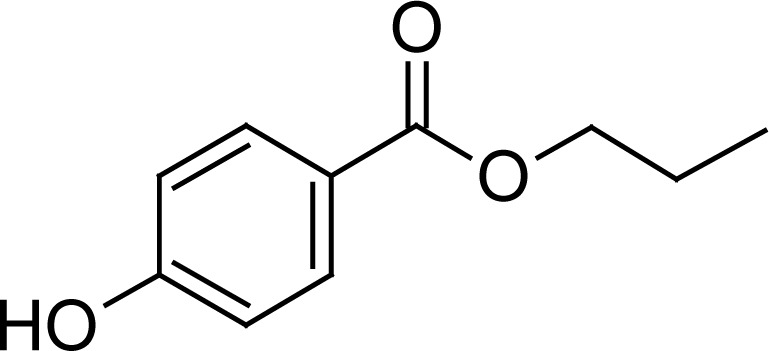

**Tab. 1.** System suitability results

**Substance**	**System suitability during Precision**			**System suitability during Intermediate Precision**		

	**Area (%RSD, n=5)**	**USP Plate count**	**USP Tailing**	**Area (%RSD, n=5)**	**USP Plate count**	**USP Tailing**

**Accept. criteria**	**≤ 2.0**	**> 5000**	**≤ 2.0**	**≤ 2.0**	**> 5000**	**≤ 2.0**
Hamometasone	0.82	13882	1.27	0.31	22115	1.14
Fusidic acid	0.57	252349	1.41	0.34	2288161	1.25
Methylparaben	0.56	15462	1.33	0.46	15717	1.22
Propylparaben	0.70	16129	1.29	0.53	17170	1.19

**Tab. 2.** Method Precision and Intermediate precision results

**Substance**	**Precision at 100%**		**Intermediate Precision at 100%**	

	**Mean % Assay**	**% RSD**	**Mean % Assay**	**% RSD**
Halometasone	100.5	0.91	101.0	1.25
Fusidic Acid	101.0	0.75	101.0	0.95
Methylparaben	100.9	0.28	99.9	0.97
Propylparaben	101.0	0.54	99.9	0.93

**Tab. 3.** Summary of forced degradation results

		**Acidic**	**Basic**	**Oxidation**	**Thermal**	**Hydrolytic**	**Photolytic**
**Hamometasone**	% Degr.	0.9	13.1	9.7	1.8	4.6	1.7
	PA	0.509	0.130	0.44	0.551	0.438	0.498
	PT	1.160	0.310	4.547	1.309	7.070	1.208

**Fusidic acid**	% Degr.	0	3.3	0.9	0	1.1	0
	PA	0.050	0.053	0.047	0.057	0.050	0.054
	PT	0.275	0.248	0.465	0.98	0.551	0.285

**Methylparaben**	% Degr.	0	7.8	4.9	1.3	2.4	0
	PA	0.040	0.032	0.047	0.055	0.052	0.060
	PT	0.306	0.220	0.721	0.315	0.325	0.313

**Propylparaben**	% Degr.	2.2	0	3.6	6.6	5.6	0.1
	PA	0.511	0.133	0.543	0.635	0.593	0.653
	PT	0.519	0.335	6.996	32.554	9.048	29.562

Degr…Degradation; PA…purity angle; PT…purity treshold. Note: Purity angle should be less then purity threshold.

**Tab. 4.** Summary of linearity data

**Parameter**	**Halometasone**	**Fusidic Acid**	**Methylparaben**	**Propylparaben**
**Linearity range (μg/mL)**	6.5–18.7	246.1–738.5	18.5–55.5	1.8–5.5
**Correlation coefficient**	0.999	0.999	0.999	0.999
**Slope**	51376	68880	26973	23368
**Intercept**	45358	68817	27427	20614

**Tab. 5.** Summary of Recovery results

**Amount spiked^a^**	**% Recovery^b^**			

	**Halometasone**	**Fusidic Acid**	**Methylparaben**	**Propylparaben**
50%	98.4 ± 0.7	98.5 ± 1.2	97.9 ± 0.2	98.6 ± 1.1
75%	98.5 ± 0.4	97.6 ± 0.1	100.2 ±0.1	99.9 ± 0.5
100%	100.8 ± 1.6	99.0 ± 0.2	100.7 ±0.4	100.8 ± 0.3
125%	99.8 ±1.9	100.6 ±1.7	100.9 ±0.3	101.0 + 1.2
150%	99.9 ± 0.9	102.6 ± 0.4	101.5 ± 0.4	102.0 ± 0.7

a Amount of all three analyte spiked with respect to target concentration.

b Mean ± %RSD for three determinations.

**Tab. 6.** Summary of Robustness results of the HPLC method

		**Column Temperature**		**Flow rate**			**M.P. Buffer**	

		**20°C**	**30°C**	**0.8 mL/min**	**1.2 mL/min**	**pH 2.3**		**pH 2.7**
**Halometasone**	*t_R_*^a^	12.715	11.652	15.542	10.474	12.841		13.067
	*A*^b^	0.28	0.25	0.32	0.34	0.99		0.46
	*T*^c^	1.30	1.31	1.23	1.31	1.30		1.30
	N^d^	11122	11702	18676	10274	11191		11254

**Fusidic Acid**	*t_R_*^a^	17.581	17.336	18.976	16.456	17.653		17.716
	*A*^b^	0.34	0.05	0.12	0.31	0.78		0.31
	*T*^c^	1.47	1.45	1.53	1.44	1.46		1.46
	N^d^	149122	142694	157296	128298	153263		160577

**Methylparaben**	*t_R_*^a^	6.071	5.731	7.501	5.017	6.061		6.114
	*A*^b^	0.22	0.07	0.08	0.38	0.46		0.22
	*T*^c^	1.40	1.38	1.41	1.37	1.39		1.39
	N^d^	11156	11370	12079	10255	11167		11182

**Propylparaben**	*t_R_*^a^	9.399	8.634	11.558	7.729	9.402		9.532
	*A*^b^	0.44	0.23	0.26	0.36	0.75		0.46
	*T*^c^	1.35	1.33	1.26	1.35	1.34		1.33
	N^d^	12295	12618	13751	11400	12128		12347

*t_R_*^a^ Retention time (min) of the analyte peak.

*A*^b^ % RSD of the analyte peak areas from 5 injections.

*T*^c^ Tailing factor of the analyte peak.

N^d^ Plate count of the analyte peak.
